# Untreated Hypertension and Diabetes in the Chest Pain Observation Unit

**DOI:** 10.5811/westjem.41560

**Published:** 2025-09-12

**Authors:** Benjamin T. Hutchison, Nicklaus P. Ashburn, Anna C. Snavely, Michael D. Shapiro, Michael A. Chado, Alexander P. Ambrosini, Amir A. Biglari, Harris A. Cannon, Marissa J. Millard, Alexa G. Dameron, Simon A. Mahler

**Affiliations:** * Wake Forest University School of Medicine, Department of Emergency Medicine, Wake Forest University, Winston-Salem, North Carolina; † Wake Forest University School of Medicine, Department of Biostatistics and Data Science, Wake Forest University, Winston-Salem, North Carolina; ‡ Wake Forest University School of Medicine, Section on Cardiovascular Medicine, Department of Internal Medicine, Wake Forest University, Winston-Salem, North Carolina; § Ohio State University, Department of Emergency Medicine, Columbus, Ohio; ¶ Wake Forest University School of Medicine, Department of Implementation Science, Wake Forest University, Winston-Salem, North Carolina; || Yale School of Medicine, Department of Internal Medicine, New Haven, Connecticut; # Wake Forest University School of Medicine, Department of Epidemiology and Prevention, Wake Forest University, Winston-Salem, North Carolina

## Abstract

**Introduction:**

Hypertension and diabetes are common cardiovascular disease risk factors among emergency department observation unit (EDOU) patients evaluated for acute coronary syndrome (ACS). Our primary aim was to determine rates of untreated hypertension and diabetes in the EDOU. Our secondary aim was to identify rates of glycemic control assessment among patients with diabetes.

**Methods:**

We conducted a retrospective, observational cohort study of patients ≥ 18 years old evaluated for ACS in a tertiary care center EDOU from March 3, 2019–February 28, 2020. Known diagnoses prior to EDOU encounter and new outpatient diagnoses within one year for hypertension and diabetes were identified by health record data. We defined untreated hypertension and diabetes as no antihypertensive or antihyperglycemic prescriptions or diabetes counseling within one year. We calculated treatment rates with exact 95% confidence intervals (CI). Multivariable logistic regression adjusting for age, sex, and race compared treatment rates among men vs women and White vs non-White patients. Rates of glycemic control assessment were defined by the proportion of patients with known diabetes who received hemoglobin A1c (HbA1c) measurement within one year.

**Results:**

Among 649 EDOU patients, 59.5% (386/649) were female and 43.8% (284/649) were non-White with a mean age of 59 ± 12 years. Of these, 76.9% (499/649) had known hypertension and 31.3% (203/649) had known diabetes. Within one year, 3.1% (20/649) had newly diagnosed hypertension and 3.2% (21/649) had newly diagnosed diabetes. Among those with known or newly diagnosed hypertension, untreated hypertension occurred in 36.4% (189/519; 95% CI 32.3 – 40.7). Hypertension treatment rates were similar in men vs women (aOR [adjusted odds ratio] 0.82, 95% CI 0.57 – 1.19) and White vs non-White patients (aOR 0.95, 95% CI 0.66 – 1.38). Among those with known or newly diagnosed diabetes, untreated diabetes occurred in 25.0% (56/224; 95% CI 18.5 – 31.2). Diabetes treatment rates were similar in men vs women (aOR 1.41, 95% CI 0.72 – 2.74) and White vs. non-White patients (aOR 1.05, 95% CI 0.56 – 1.97). At one year, just 32.0% (65/203) of patients with diabetes had HbA1c testing.

**Conclusion:**

Given that many patients evaluated for acute coronary syndrome in the ED observation unit do not receive treatment for hypertension and diabetes within one year of presentation, clinicians should consider initiating EDOU-based preventive cardiovascular care for these conditions.

## INTRODUCTION

Hypertension and diabetes are independent risk factors for atherosclerotic cardiovascular disease (ASCVD) and represent substantial causes of all-cause mortality and healthcare costs in the United States. Hypertension affects up to 46% of American adults and is the most prevalent cardiovascular risk factor worldwide.^1^,^2^ It is also the single most significant and modifiable risk factor for stroke and cardiovascular disease.^3^ Diabetes represents another consequential chronic disease and is the leading cause of preventable deaths in the US, currently affecting over 38 million Americans.^1^,^4^

Due to their strong association with ASCVD, hypertension and diabetes are commonly found in patients presenting to the emergency department (ED) with acute chest pain, which accounts for > 7.0 million ED visits per year.^5^–^9^ An estimated 25 million patients present to the ED with asymptomatic hypertension each year, but < 4% are discharged with antihypertensive therapy.^10^ Given the strong association of hypertension with all-cause mortality, the American College of Emergency Physicians (ACEP) guidelines recommend considering initiation of antihypertensive medication for asymptomatic hypertension in select patients, although this practice is rarely followed by emergency clinicians.^10^,^11^

Similarly, while some have recommended formally diagnosing and treating new diabetes in the ED, there is no widely accepted practice recommendation supporting these practices.^9^,^12^,^13^ Therefore, management of new or uncontrolled asymptomatic hypertension or diabetes in the ED is often viewed as an outpatient condition and rarely addressed in the acute care setting. Although emergency clinicians often advise these patients to follow up with their primary care physician for further management, many patients fail to receive appropriate outpatient care in a timely manner after ED discharge, including the recommended twice yearly hemoglobin A1c (HbA1c) testing of those with diabetes.^14^,^15^ Thus, there may be an opportunity for emergency clinicians to initiate preventive cardiovascular care for patients presenting with acute chest pain in the ED and ED observation units (EDOU) by treating hypertension and diabetes.

To assess the possible impact of EDOU-initiated preventive cardiovascular care for hypertension and diabetes, we evaluated a cohort of patients presenting with acute chest pain to the ED/EDOU of a large, academic, tertiary-care center. Our primary aim was to evaluate rates of untreated hypertension and diabetes in the EDOU. We hypothesized that while many patients evaluated in the EDOU would have hypertension and diabetes, most would not receive treatment for their known hypertension or diabetes in the EDOU or within one year after EDOU discharge. We also assessed the proportion of patients without a previous diagnosis of hypertension or diabetes who received treatment for newly diagnosed hypertension and diabetes within the one-year follow-up period. As a secondary aim, we assessed rates of glycemic control testing in patients with known diabetes by determining whether these patients received HbA1c measurement in the EDOU or within one year of EDOU encounter. Given known disparities in cardiovascular care among women and non-White patients, we also evaluated for potential disparities in the treatment of these conditions and for glycemic control evaluations among patients with diabetes.^16^–^20^

**Population Health Research Capsule**

What do we already know about this issue?

*Hypertension and diabetes are common risk factors for cardiovascular disease among patients evaluated for acute coronary syndrome (ACS) in the ED observation unit (EDOU)*.

What was the research question?

*Among those evaluated for ACS in the EDOU, what is the 1-year treatment rate for hypertension and diabetes?*

What was the major finding of the study?

*Over one year, untreated hypertension occurred in 36.4% and untreated diabetes occurred in 25.0%*.

How does this improve population health?

*Many patients in the EDOU have untreated hypertension and diabetes within one year of presentation, suggesting possible opportunities to initiate EDOU-based care*.

## METHODS

### Study Design

We conducted a retrospective, observational, cohort study of patients presenting with acute chest pain and evaluated for possible acute coronary syndrome (ACS) in the EDOU of a large, academic teaching hospital from March 1, 2019 – February 28, 2020 by leveraging the Wake Forest EDOU Chest Pain Registry. The Wake Forest University Health Sciences Institutional Review Board reviewed and approved the study protocol and granted a waiver of informed consent for the study. The Strengthening the Reporting of Observational Studies in Epidemiology (STROBE) guidelines helped direct the research and reporting processes.^21^

### Study Setting and Population

The study included patients ≥ 18 years of age being evaluated for possible ACS in the EDOU at Atrium Health Wake Forest Baptist, a large, academic teaching hospital in North Carolina. The study cohort was comprised of a convenience sample of consecutive patients. The EDOU is a type 1, protocol-driven observation unit and is managed by emergency physician assistants and nurse practitioners who are supervised by board-certified or board-eligible emergency physicians. Patients with ST-segment elevation myocardial infarction, hemodynamic instability (heart rate < 40 or > 120 beats per minute, systolic blood pressure < 90 millimeters of mercury mmHg, or oxygen saturation SpO2% < 90% on room air or normal home oxygen flow rate), high-sensitivity cardiac troponin I (Beckman Coulter Diagnostics Brea, CA) ≥ 100 nanograms per liter, or trauma were not eligible for EDOU care and, thus, were excluded from the study. The EDOU chest pain protocol is available in Supplemental Appendix A. Per the EDOU chest pain protocol, patients were evaluated for potential ACS with serial troponin measurements, telemetry, and possible stress testing or coronary computed tomography angiography. During the study, no specific additional training or guidance was provided to clinicians in the EDOU regarding hypertension or diabetes management.

### Data Collection and Variables

Index encounter data (from initial ED presentation to discharge from the EDOU) through one year of outpatient follow-up were abstracted from the electronic health record (EHR) (Epic Systems Corporation, Verona, WI) by trained data abstractors and entered into the Wake Forest EDOU Chest Pain Registry. Outpatient follow-up included any primary care or specialty clinic visits captured in the EHR system for our health system network. All network hospitals and outpatient clinics share the same EHR, and the network is the largest in the region, making it likely to capture the vast majority of follow-up visits for patients in the study. We previously performed insurance claims analysis of our network EHR, which found very few events at outside network hospitals, confirming the reliability of in-network EHR follow-up visit rates.^22^

Hypertension and diabetes diagnoses, sex, race, and medication use were identified by patient self-report, *International Classification of Diseases, 10^th^ Rev*, ICD-10 codes, and/or clinician documentation in the EHR. Initiation of antihypertensive medication, diabetes treatment, and/or measurement of HbA1c in the EDOU and future outpatient visits were also abstracted from the EHR. Data were entered into a Research Electronic Data Capture (REDCap) database hosted at Wake Forest University School of Medicine. We followed best practice guidelines for data abstraction, including data abstractor training and optimizing case selection criteria using variable definitions, as well as a data dictionary and standardized digital abstraction forms within REDCap.^23^,^24^ Data abstractor performance was monitored throughout the study period.^23^,^24^ Training included in-person instruction with the principal investigator (PI) where patient encounters and data were reviewed to ensure familiarity with accessing relevant data in the EHR and inputting data into REDCap. Additionally, the PI reviewed a random sample of entries to ensure accuracy.

### Outcomes

Study outcomes were 1) the proportion of patients with known or newly diagnosed hypertension who received antihypertensive treatment at their index EDOU visit and within the one-year follow-up period, and 2) the proportion of patients with known or newly diagnosed diabetes who received diabetes treatment at their EDOU index visit and within the one-year follow-up period. New diagnoses of hypertension and diabetes were defined by patients with new diagnoses by outpatient clinician documentation or ICD-10 code during the one-year follow-up period. We defined untreated hypertension as a patient with known or newly diagnosed hypertension who did not receive antihypertensive treatment at their index EDOU visit or within one year of follow-up. Untreated diabetes was defined as a patient with known or newly diagnosed diabetes who did not receive diabetes treatment at their index visit or within one year of follow-up. Consistent with prior studies, patients without documentation of one-year follow-up for either testing or treatment in the EHR (i.e., those “lost to follow-up”) were assumed to not have received care.

We defined antihypertensive treatment as any blood pressure-lowering agent, such as an angiotensin-converting-enzyme inhibitor, angiotensin-receptor blocking agent, beta blocker, calcium channel blocker, hydralazine, or diuretic (including loop diuretic, thiazide diuretics, and potassium-sparing diuretics). Diabetes treatment was defined as the composite of any anti-hyperglycemic drug treatment (insulin, metformin, dipeptidyl peptidase-4 inhibitor, or glucagon-like peptide-1 receptor antagonists) or documentation of diabetes counseling. A secondary outcome measure was rates of glycemic control assessment, defined as having an HbA1c measurement in the EDOU or in the one-year follow-up period among patients with a known diagnosis of diabetes.

### Statistical Analysis

We used counts, percentages, and means with standard deviations to describe the study population. Rates of antihypertensive treatment, diabetes treatment (including drug treatment and counseling), and diabetes evaluation (HbA1c testing) during the index EDOU encounter and the one-year follow-up period inclusive of the index EDOU encounter were calculated and reported along with exact 95% confidence intervals (CI). We used Fisher exact test to compare rates of hypertension treatment, diabetes treatment, and diabetes evaluation at one year among White vs non-White patients and men vs women. To further evaluate the association of race and sex with hypertension and diabetes treatment at one year, we performed multivariable logistic regression adjusting for age (continuous), race (White vs non-White), and sex (male vs female). Unadjusted and adjusted odds ratios (aOR) with corresponding 95% CIs were calculated.

## RESULTS

**Figure 1:**
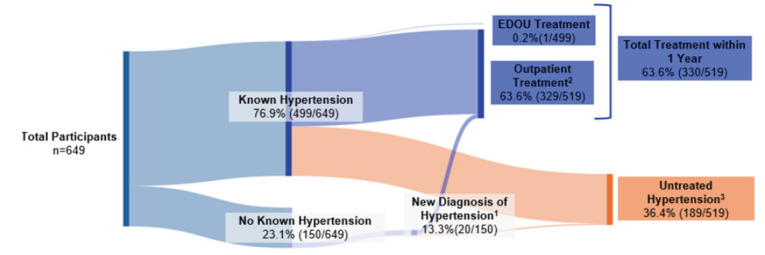
Untreated hypertension in the emergency department observation unit. ^1^ New diagnoses of hypertension made at outpatient visits during the 1-year follow-up period of 150 of patients with no known hypertension prior to EDOU encounter. ^2^ Treatment for hypertension at outpatient visit within 1 year of EDOU encounter of 519 of patients with known or new diagnoses of hypertension. ^3^ No treatment in EDOU or within 1 year of 519 of patients with known or new diagnoses of hypertension, *EDOU*, emergency department observation unit.

**Figure 2:**
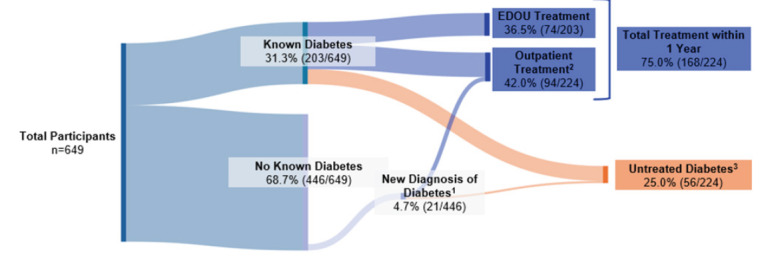
Untreated diabetes in the emergency department observation unit. ^1^ New diagnoses of diabetes made at outpatient visits during the 1-year follow-up period of 446 of patients with no known diabetes prior to EDOU encounter. ^2^ Treatment for diabetes at outpatient visit within 1 year of EDOU encounter out of n=224 of patients with known or new diagnoses of diabetes, ^3^ No treatment in EDOU or within 1 year of 224 of patients with known or new diagnoses of diabetes. *EDOU*, emergency department observation unit.

**Figure 3:**
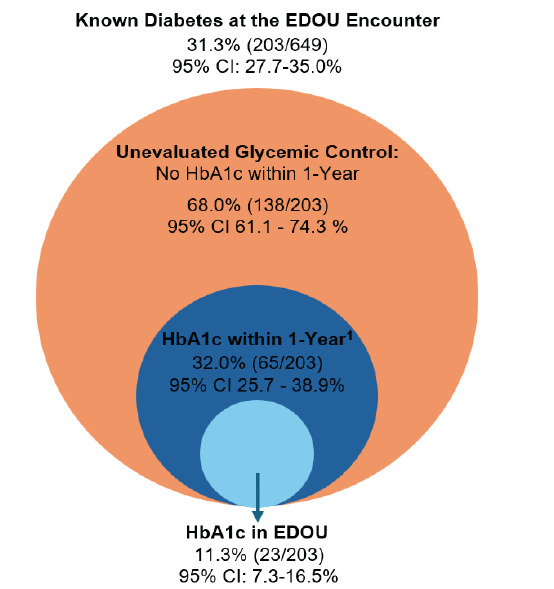
Rates of HbA1c testing in the emergency department observation unit among patients with known diabetes. ^1^ HbA1c measurement in EDOU or outpatient clinic visit within 1 year of EDOU encounter. *EDOU*, emergency department observation unit; *HbA1c*, hemoglobin A1C; *CI*, confidence interval.

During the study period, 649 patients were evaluated in the EDOU for possible ACS, of whom 59.5% (386/649) were female and 43.8% (284/649) were non-White with a mean age of 59.8 ± 12.3 years. Among these patients, 76.9% (499/649) had known hypertension and 31.3% (203/649) had known diabetes. During the one-year follow-up period, 69.7% (452/649) were evaluated in an outpatient clinic for any reason. Newly diagnosed hypertension occurred in 13.3% (20/150) of patients without a previous diagnosis of hypertension, and newly diagnosed diabetes occurred in 4.7% (21/446) of patients without a previous diagnosis of diabetes. Table 1 describes the cohort characteristics. Untreated hypertension occurred in 36.4% (189/519; 95% CI 32.3 – 40.7) of patients with known or newly diagnosed hypertension. These patients did not receive any form of antihypertensive treatment while in the EDOU or during the one-year follow-up period (Figure 1). In addition, untreated diabetes was observed in 25.0% (56/224; 95% CI 18.5 – 31.2) of patients with known or newly diagnosed diabetes. These patients did not receive any type of antihyperglycemic treatment or diabetes counseling while in the EDOU or during the one-year follow-up period (Figure 2). Differences in treatment rates for hypertension and diabetes among those with known or new diagnoses are summarized in Table 2. Finally, rates of glycemic control assessment among diabetics were low, as just 32.0% (65/203) of patients with known diabetes had a HbA1c measurement within one year of their EDOU encounter (Figure 3). Furthermore, only 11.3% (23/203) of patients with known diabetes received HbA1c evaluation in the EDOU. For hypertension, treatment rates at one year were similar in White vs. non-White patients (63.2% [172/272] vs 64.0% [158/247]; *P* = .93) and in men vs women (60.2% [124/206] vs 65.8% [206/313]; *P* = .23). The rate of diabetes treatment at one year was also similar in White vs non-White patients (75.5% [74/98] vs 74.6% [94/126], *P* = 1.0) and in men vs women (79.8% [67/84] vs 72.1% [101/140], *P* = .26). After adjusting for potential confounders, hypertension and diabetes treatment rates remained similar between White vs non-White patients and between men vs women. Table 3 represents rates of hypertension treatment among race and sex subgroups, while Table 4 describes rates of diabetes treatment among race and sex subgroups.

## DISCUSSION

This study demonstrates substantial rates of untreated hypertension and diabetes and reveals the potential for ED-initiated preventive cardiovascular care among patients with acute chest pain evaluated in an EDOU. Approximately 80% of chest pain observation patients had hypertension, and over one-third had diabetes. Despite the high prevalence of these key cardiovascular disease risk factors, many did not receive appropriate medical therapy within one year of their emergency care encounter. Even fewer patients received preventive care while in the EDOU. These findings suggest that we may be missing an opportunity to impact long-term health outcomes in patients during their EDOU visit.

Given the enormous impact that hypertension and diabetes treatment have on ASCVD risk and overall mortality, the US Department of Health and Human Services aims to improve cardiovascular health with the Healthy People 2030 initiative, specifically encouraging advancements in adult hypertension control and ASCVD risk assessment.^25^ Lowering blood pressure and controlling glucose levels in patients with hypertension and diabetes reduces rates of cardiovascular disease and improves mortality.^2^,^26^–^31^ While initiating appropriate preventive cardiovascular care can have life-saving effects, current practice guidelines regarding this practice in the ED are limited and not uniformly followed.

Many patients with hypertension and diabetes may not receive guideline-directed care in the outpatient setting after ED discharge, which we redemonstrated in our EDOU-specific study.^31^–^34^ Although ACEP guidelines encourage emergency clinicians to consider providing lifestyle counseling and initiating low-dose pharmacotherapy in select high-risk groups in the ED, there is limited literature to inform current practice in the ED and EDOU.^11^,^35^ While initiating preventive care from the ED is not currently standard practice, the high rates of patients who are unable to receive outpatient care in the outpatient setting may highlight an opportunity to initiate potentially life-saving preventive care in the ED setting. We recommend further study and discussion to improve practice guidelines for preventive care in the EDOU.

There may be barriers to implementing preventive cardiovascular care in the ED and EDOU. Potential barriers to ED-initiated preventive care include the loud, busy, and chaotic nature of the ED, which is not always conducive to preventive care practices such as lifestyle modification counseling. Furthermore, staff constraints, limited resources, and the presence of other high-acuity emergencies may also pose barriers to initiating care for hypertension and diabetes. Amidst these barriers, prior studies have highlighted the challenge for emergency clinicians to address public health concerns and preventive medicine.^36^ The EDOU may, however, be more conducive to implementing preventive care for these comorbidities. In the EDOU, patients can more easily receive serial blood pressure measurements, further diagnostic testing like HbA1c, and effective lifestyle counseling. Future work and implementation studies are needed to develop high-impact, evidence-based medical decision-making frameworks and best practice recommendations to help emergency clinicians deliver preventive care.

Data are limited on the use of the EDOU for treatment of hypertension and diabetes. While some studies have assessed the prevalence of asymptomatic hypertension and diabetes in the ED, few have examined the impact of treating these conditions in the ED or EDOU on patient-centered outcomes.^8^,^10^,^37^,^38^ A recent expert opinion review provided recommendations for hypertension management in the EDOU and emphasized the feasibility of accurate diagnosis and initiation of treatment in the EDOU.^34^ While a recent randomized controlled trial evaluated management of asymptomatic hypertension in the ED and demonstrated feasibility for ED-based treatment, this was in the ED setting rather than an observation unit.^39^ The data for diabetes treatment in the ED or EDOU are even more sparse, with no high-quality or clinical trial evidence to help guide emergency clinicians. Our own research group has previously described other untreated cardiovascular conditions in the EDOU and has highlighted the potential for emergency clinicians to initiate potentially life-saving therapies such as smoking cessation and lipid-lowering medications.^40^–^43^ Together, these findings suggest that the EDOU may be an appropriate location for initiating comprehensive preventive cardiovascular care for multiple cardiovascular disease risk factors.

As we explored rates of glycemic control assessments among patients with known diabetes, we found there may be an opportunity to improve diabetes testing in the EDOU. Current guidelines recommend HbA1c testing at least twice per year in patients with known diabetes.^44^,^45^ While many patients being evaluated for ACS in the EDOU have known diabetes, just 32% of these patients were evaluated for diabetes within one year of their EDOU encounter, suggesting there may be a possible opportunity for EDOU-based glycemic control evaluation in some patients being evaluated for ACS.^46^ Additional studies are needed to further explore this potential missed opportunity.

Importantly, our study did not find significant differences in hypertension and diabetes treatment among sex or race groups. Of note, the rate of diabetes treatment within one year of the index EDOU visit was 8% higher among men than women. While not statistically significant, this could have been due to our cohort’s modest sample size. Given this finding and the ample literature highlighting disparities in diabetes and hypertension care among men and women, future ED and EDOU preventive cardiovascular care disparities research is warranted.^16^–^20^

## LIMITATIONS

This study has limitations. While the study was conducted at one academic center and focused on EDOU patients, many EDs now operate protocol-driven EDOUs. Although this study was retrospective, best practices for retrospective review were used to enhance accuracy and scientific rigor. It is possible that patients may have had outpatient clinic visits or treatment initiated at outside medical systems not available in our EHR, thereby introducing a potential source of misclassification bias. However, our health system is the largest in the region with all network hospitals and outpatient clinic sites sharing the same EHR.

While our study was able to evaluate for rates of glycemic control assessments by measuring rates of HbA1C ordering in diabetics, we were unable to similarly assess blood pressure control assessments in patients with known hypertension, as we did not have access to all blood pressure measurements within one year. However, we did assess new diagnoses of hypertension as reported by clinician documentation or EHR data. Similarly, for patients initiated on hypertension or diabetes treatment, we did not monitor treatment adherence over one year. While the study adhered to most best practices for optimal chart review, our data abstractors were not blinded to the study hypothesis, we did not report on interrater reliability, and we did not formally measure percentage agreement or Kappa.^24^ Lastly, the precision of our study was limited by the modest sample size.

## CONCLUSION

This study highlights high rates of untreated hypertension and diabetes in the emergency department observation unit. Because large numbers of patients with hypertension and diabetes did not receive appropriate treatment in the outpatient setting, the EDOU may be an appropriate place to bridge this gap in care. Similarly, most patients with diabetes did not receive an adequate glycemic control evaluation within one year, so there may be opportunity for EDOU-based HbA1c assessments in these patients. Given the potential life-saving risk reduction associated with hypertension and diabetes management, emergency clinicians may be able to help mitigate mortality by initiating proper treatment in the EDOU. We recommend additional investigation with pilot intervention models in the EDOU to assess the feasibility and effectiveness of EDOU-initiated hypertension and diabetes treatment programs.

## Supplementary Information



## Figures and Tables

**Table 1:** Cohort characteristics of patients with known and new diagnoses of hypertension and diabetes.

Patient characteristics	No known hypertension n = 150, (%)	Known hypertension n = 499, (%)	No known diabetes n = 446, (%)	Known diabetes n = 203, (%)	Total N = 649, (%)
Age (mean ±SD) (years)	57.0 (12.5)	60.6 (12.1)	59.8 (12.8)	59.6 (11.1)	59.8 (12.3)
Sex					
Female	83 (55.3)	196 (39.3)	259 (58.1)	127 (62.6)	386 (59.5)
Race					
White	106 (70.1)	259 (51.9)	276 (61.9)	89 (43.8)	365 (56.2)
Black	24 (16.0)	200 (40.1)	140 (31.4)	84 (41.4)	224 (34.5)
Other	20 (13.3)	40 (8.0)	30 (6.7)	30 (14.8)	60 (9.2)
Ethnicity					
Hispanic or Latino	15 (10.0)	28 (5.6)	23 (5.2)	20 (9.9)	43 (6.6)
Comorbidities					
Obesity (BMI ≥ 30 kg/m^2^)	44 (29.3)	301 (60.3)	210 (47.1)	135 (66.5)	345 (53.2)
Hypertension	0%	100%	316 (70.9)	183 (90.2)	499 (76.9)
Diabetes	20 (13.3)	183 (36.7)	0%	100%	203 (31.3)
Known CAD	7 (4.7)	74 (14.8)	52 (11.7)	29 (14.3)	81 (12.5)
Stroke	10 (6.7)	58 (11.6)	40 (9.0)	28 (13.8)	68 (10.5)
HLD	54 (36.0)	308 (61.7)	217 (48.6)	145 (71.4)	362 (55.8)
CKD	3 (2.0)	60 (12.0)	34 (7.6)	29 (14.3)	63 (9.7)

*BMI*, body mass index; *CAD*, coronary artery disease; *HLD*, hyperlipidemia; *CKD*, chronic kidney disease.

**Table 2:** Treatment rates of patients with known and new diagnoses of hypertension and diabetes.

	Known hypertension n = 499, (%)	New hypertension diagnosis^1^ n= 20, (%)	Known diabetes n = 203, (%)	New diabetes diagnosis^1^ n = 21, (%)
Untreated Condition^2^	184 (36.9)	5 (25.0)	49 (24.1)	7 (33.3)
Treatment in the EDOU	1 (0.2)	N/A^3^	74 (36.5)	N/A^3^
Treatment within 1 Year	314 (62.9)	15 (75.0)	80 (39.4)	14 (66.7)

1 New diagnoses of hypertension or diabetes made at outpatient visits during the 1-year follow-up period.

2 No treatment in EDOU or within 1 year.

3 Patients with new diagnoses of hypertension or diabetes within the 1-year follow-up period did not receive treatment while in the EDOU.

*EDOU*, emergency department observation unit.

**Table 3:** Rates of hypertension treatment among patients with known and newly diagnosed hypertension by race (White vs non-White) and sex (male vs. female).

	White n = 272, (%)	Non-White n = 247, (%)	Unadjusted OR (95% CI)	Adjusted OR^1^ (95% CI)
Index EDOU	0 (0.0)	1 (0.4)	N/A 3	N/A 3
EDOU through 1-Year Follow-up	172 (63.2)	158 (64.0)	0.97 (0.68–1.39)	0.95 (0.66–1.38)

	Male n = 206, (%)	Female n = 313, (%)	Unadjusted OR (95% CI)	Adjusted OR^2^ (95% CI)
Index EDOU	0 (0.0)	1 (0.3)	N/A 3	N/A 3
EDOU through 1-Year Follow-up	124 (60.2)	206 (65.8)	0.79 (0.55–1.13)	0.82 (0.57–1.19)

1 djusted for age (continuous) and sex (male vs female).

2 djusted for age (continuous) and race (White vs non-White).

3 nable to adjust due to the lower number of events.

*EDOU*, emergency department observation unit; *OR*, odds ratio; *CI*, confidence interval.

**Table 4:** Rates of diabetes treatment among patients with known and newly diagnosed diabetes by race (White vs non-White) and sex (male vs. female).

	White n = 98, (%)	Non-White n = 126, (%)	Unadjusted OR (95% CI)	Adjusted OR^1^ (95% CI)
Index EDOU	33 (33.7)	45 (35.7)	0.91 (0.52–1.59)	0.94 (0.53–1.66)
EDOU through 1-Year Follow-up	74 (75.5)	94 (74.6)	1.05 (0.57–1.93)	1.05 (0.56–1.97)

	Male n = 84, (%)	Female n = 140, (%)	Unadjusted OR (95% CI)	Adjusted OR^2^ (95% CI)
Index EDOU	29 (34.5)	49 (35.0)	0.98 (0.56–1.73)	0.96 (0.54–1.73)
EDOU through 1-Year Follow-up	67 (79.8)	101 (72.1)	1.52 (0.80–2.91)	1.41 (0.72–2.74)

1 djusted for age (continuous) and sex (male vs female).

2 djusted for age (continuous) and race (White vs non-White).

*EDOU*, emergency department observation unit; *OR*, odds ratio; *CI*, confidence interval.
