# Supplementary figures and images for: Untreated Hypertension and Diabetes in the Chest Pain Observation Unit
Source: West J Emerg Med. 2025 Sep 12;26(5):1296–304. doi: 10.5811/westjem.41560 (PMC12591649; doi:10.5811/westjem.41560)

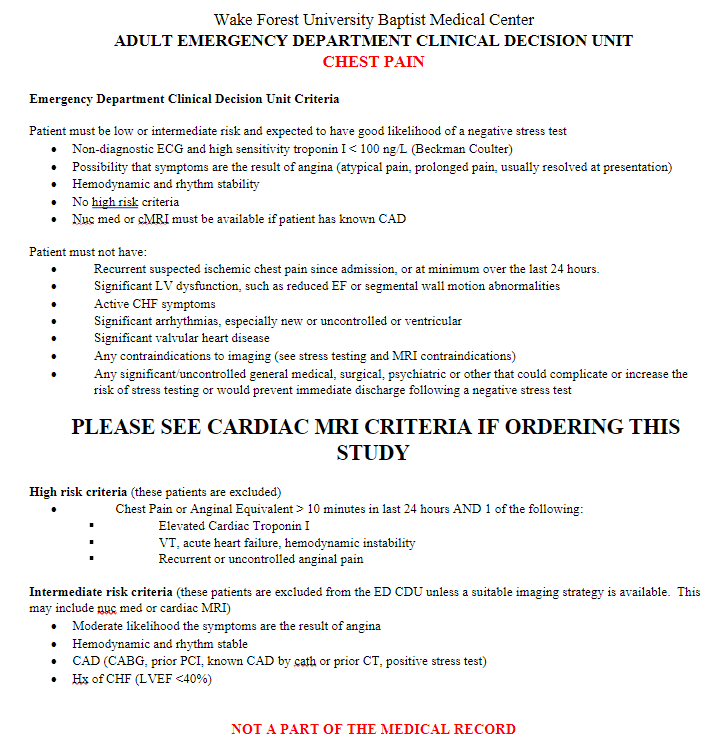

Supplement: Supplementary file 1 [file wjem-26-1296-s001.docx]
